# Trypanocidal and leishmanicidal activity of six limonoids

**DOI:** 10.1007/s11418-020-01408-7

**Published:** 2020-04-10

**Authors:** Dietmar Steverding, Lazare S. Sidjui, Éden Ramalho Ferreira, Bathelemy Ngameni, Gabriel N. Folefoc, Valérie Mahiou-Leddet, Evelyne Ollivier, G. Richard Stephenson, Thomas E. Storr, Kevin M. Tyler

**Affiliations:** 1 Bob Champion Research and Education Building, Norwich Medical School, University of East Anglia, Norwich, NR4 7UQ UK; 2 Institute of Medical Research and Medicinal Plant Studies, P.O. Box 13033, Yaoundé, Cameroon; 3 Bioorganic and Medicinal Chemistry Laboratory, Department of Organic Chemistry, Faculty of Sciences, University of Yaoundé I, Yaoundé, Cameroon; 4 BioMedical Research Centre, Norwich Medical School, University of East Anglia, Norwich, NR4 7TJ UK; 5 Departamento de Microbiologia, Imunologia e Parasitologia, Escola Paulista de Medicina, Universidade Federal de São Paulo, São Paulo, Brazil; 6 Department of Pharmacognosy and Pharmaceutical Chemistry, Faculty of Medicine and Biomedical Science, University of Yaoundé I, Yaoundé, Cameroon; 7 Aix-Marseille University, Avignon University, CNRS, IRD, IMBE, FAC PHARM, Marseille, France; 8 School of Chemistry, University of East Anglia, Norwich, NR4 7TJ UK

**Keywords:** Limonoids, African trypanosomiasis, *Trypanosoma brucei*, Leishmaniasis, *Leishmania major*

## Abstract

Six limonoids [kotschyienone A and B (**1**, **2**), 7-deacetylgedunin (**3**), 7-deacetyl-7-oxogedunin (**4**), andirobin (**5**) and methyl angolensate (**6**)] were investigated for their trypanocidal and leishmanicidal activities using bloodstream forms of *Trypanosoma brucei* and promastigotes of *Leishmania major*. Whereas all compounds showed anti-trypanosomal activity, only compounds **1–4** displayed anti-leishmanial activity. The 50% growth inhibition (GI_50_) values for the trypanocidal and leishmanicidal activity of the compounds ranged between 2.5 and 14.9 μM. Kotschyienone A (**1**) was found to be the most active compound with a minimal inhibition concentration (MIC) value of 10 μM and GI_50_ values between 2.5 and 2.9 μM. Only compounds **1** and **3** showed moderate cytotoxicity against HL-60 cells with MIC and GI_50_ values of 100 μM and 31.5–46.2 μM, respectively. Compound **1** was also found to show activity against intracellular amastigotes of *L. major* with a GI_50_ value of 1.5 μM. The results suggest that limonoids have potential as drug candidates for the development of new treatments against trypanosomiasis and leishmaniasis.

**Electronic supplementary material:**

The online version of this article (10.1007/s11418-020-01408-7) contains supplementary material, which is available to authorized users.

## Introduction

Trypanosomiasis and leishmaniasis are devastating diseases for both humans and their domestic animals. Trypanosome parasites cause sleeping sickness in humans and nagana disease in cattle in Africa and Chagas disease in humans in Latin America [1, 2]. The different *Leishmania* parasites cause a variety of clinical conditions (localised skin lesions, mucosal ulcers, and internal organ damage) in humans worldwide [3]. These parasites are kinetoplastid protozoans and are transmitted to their mammalian host by insect vectors. Treatment of these parasitoses relies on chemotherapy but only a few drugs are available. However, most of the drugs are not well tolerated or show toxic side effects, are not very effective, and are being increasingly subject to drug resistance. Therefore, effective and better-tolerated chemotherapies are urgently needed for the treatment of trypanosomiasis and leishmaniasis.

Many approved drugs are based on natural compounds and their derivatives [4]. In addition, a considerable number of natural products have been shown to display potent anti-trypanosomal and anti-leishmanial activity [5]. Plants that are used in traditional medicine are promising starting materials for the discovery of natural compounds with trypanocidal and leishmanicidal activity. One such plant is *Pseudocedrela kotschyi* (Schweinf.) Harms (Meliaceae), which is used for the treatment of trypanosomiasis in the Kaduna state of Nigeria [6]. Furthermore, dichloromethane extracts of the roots of *P. kotschyi* have been shown to display anti-leishmanial activity against intracellular amastigotes of *L. major* [7].

**Fig. 1:**
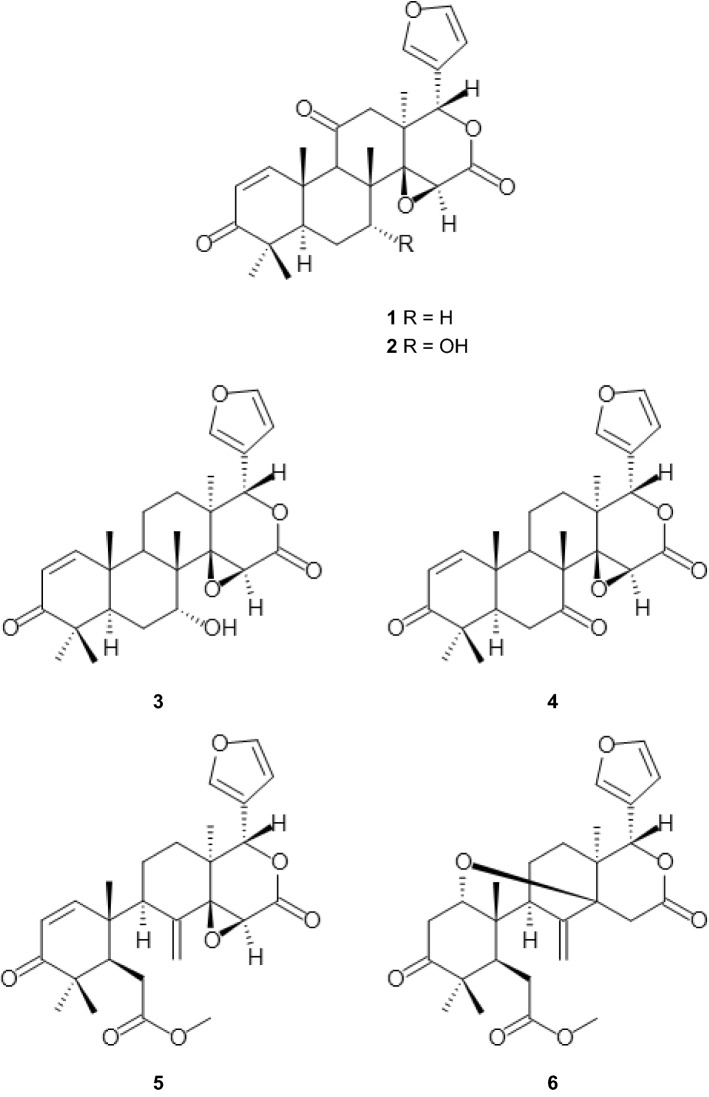
Structures of compounds **1–6**. Kotschyienone A (**1**), kotschyienone B (**2)**, 7-deacetylgedunin (**3**), 7-deacetyl-7-oxogedunin (**4**), andirobin (**5**), methyl angolensate (**6**)

In this study, we investigated the trypanocidal and leishmanicidal activity of six limonoids (Fig. 1, compounds **1–6**) isolated from the roots of *P. kotschyi*.

## Experimental

### Compounds

The limonoids **1–6** were isolated from the roots of *P. kotschyi* as previously described [8]. The compounds have been characterized by NMR (kotschyienone A and B (**1**, **2** [8], 7-deacetylgedunin (**3**) [9, 10], 7-deacetyl-7-oxogedunin (**4**) [9, 11], andirobin (**5**) [12] and methyl angolensate (**6**) [11]) and their purity was judged to be about 95% by TLC. The ^1^H-NMR and ^13^C-NMR spectra of **1**–**6** are shown in Supplementary Figures S1–6.

### Cell culture

Bloodstream forms of *Trypanosoma brucei* (clone 427-221a [13]) and human myeloid leukaemia HL-60 cells [14] were maintained in Baltz medium [15] supplemented with 16.7% heat-inactivated bovine serum in a humidified atmosphere containing 5% CO_2_ at 37 °C. Promastigotes of *Leishmania major* (strain MHOM/IL/81/Friedlin [16]) were cultured in Schneider’s insect medium supplemented with 10% heat-inactivated foetal bovine serum in a humidified atmosphere containing 5% CO_2_ at 27 °C.

### In vitro toxicity assay

Toxicity assays were carried out as previously described with some modifications [17, 18]. In brief, cells were seeded in 96-well plates in a final volume of 200 μL of appropriate medium containing various concentrations of test compounds (tenfold dilution from 100 µM to 1 nM) and 1% DMSO. Wells just containing medium and 1% DMSO served as controls. The initial cell densities were 1 × 10^4^/mL for *T. brucei* bloodstream forms, 2.5 × 10^5^/mL for *L. major* promastigotes, and 5 × 10^4^/mL for HL-60 cells. After 24 h incubation, 20 µL of a 0.5 mM resazurin solution prepared in PBS was added and the cells were incubated for a further 48 h. Thereafter, the absorbance of wells was read on a BioTek ELx808 microplate reader using a test wavelength of 570 nm and a reference wavelength of 630 nm. The 50% growth inhibition (GI_50_) value, i.e., the concentration of a compound necessary to reduce the growth rate of cells by 50% compared to the control, was determined by linear interpolation [19]. The minimum inhibitory concentration (MIC) values, i.e. the concentration of a compound at which all trypanosomes and human cells were killed, was determined microscopically.

### Intra-macrophages amastigote assay

The intra-macrophages amastigote assay was performed as previously described with some modification [20]. In brief, 1 mL of J774 cells (7 × 10^4^) suspended in RPMI supplemented with 10% foetal bovine serum were pipetted into wells of a 24 well plate containing sterile glass coverslips. Subsequently, cells were incubated overnight at 37 °C and 5% CO_2_ in a humidified incubator. The next day, metacyclic *L. major* promastigotes (MOI 20:1) were added and the plates incubated for 24 h at 34 °C and 5% CO_2_ in a humidified incubator. After 24 h incubation, cells were washed 4 times with PBS to remove parasites that had not invaded any macrophage. Then, cells were incubated with compound **1** at the following concentrations: 10, 2.5, 1.25, 0.625 and 0.1 µM. Amphotericin B (0.1 µM) was used as a positive control and no drug treatment as a negative control. After 72 h of drug incubation, cells were gently washed 4 times with PBS and fixed with 4% paraformaldehyde for 30 min. Thereafter, cells were washed once with water and stained with Giemsa for 40 min. Cells on coverslips were then destained using the acetone-xylene protocol as follows: first, pure acetone; second, pure acetone; third, acetone/xylene 9:1; forth, acetone/xylene 7:3; fifth, acetone/xylene 3:7; sixth, pure xylene. Finally, coverslips were mounted on slides with Entellan new mounting medium. Infection index (parasites/cell) were determined by counting approximately 300 cells per coverslips using an Olympus BX50 microscope with a 100 × N.A. 1.35 oil immersion objective.

## Results and discussion

All six limonoids **1–6** showed a dose-dependent inhibitory effect on the growth of bloodstream forms of *T. brucei* with MIC values ranging from 10 to > 100 μM and GI_50_ values ranging from 2.48 to 14.5 μM (Table 1). Similar activities were also observed with promastigotes of *L. major* for the four gedunin-type limonoids **1**–**4** (Table 1). In contrast to their trypanocidal activity, the two andirobin-type limonoids **5** and **6** displayed no leishmanicidal activity (MIC and GI_50_ > 100 μM, Table 1). The most active compound against both parasites was **1** with a MIC value of 10 μM and GI_50_ value of 2–3 μM (Table 1). On the other hand, only compounds **1** and **3** showed some cytotoxic activity against human HL-60 cells with MIC values of 100 μM and GI_50_ values in the mid micromolar range (Table 1). The other four compounds (**2**, **4**, **5** and **6**) displayed no cytotoxicity against HL-60 cells (MIC and GI_50_ > 100 μM, Table 1). Based on the antiparasitic and cytotoxic activities, only the gedunin-type limonoid **1** exhibited moderated selectivity indices with MIC and GI_50_ ratios of ≥ 10 (Table 2).

**Table 1:** In vitro trypanocidal, leishmanicidal and cytotoxic activity of the limonoids **1–6**

Compound	*T. brucei*		*L. major*		HL-60 cells	
	MIC (μM)	GI_50_ (μM)	MIC (μM)	GI_50_ (μM)	MIC (μM)	GI_50_ (μM)
**1**	10	2.48 ± 0.12	10	2.86 ± 0.89	100	31.5 ± 3.7
**2**	100	14.2 ± 2.9	100	14.9 ± 5.9	> 100	> 100
**3**	100	14.5 ± 3.2	100	11.6 ± 1.6	100	46.2 ± 6.7
**4**	> 100	3.18 ± 0.48	> 100	7.63 ± 2.78	> 100	> 100
**5**	> 100	11.5 ± 6.3	> 100	> 100	> 100	> 100
**6**	> 100	6.04 ± 2.25	> 100	> 100	> 100	> 100
Suramin	1	0.042 ± 0.004	nt	nt	> 100	> 100
Amphotericin B	nt	nt	0.1	0.036 ± 0.001	> 100	50.7 ± 9.3

*nt* not tested

**Table 2:** Selectivity indices of the limonoids **1–6**

Compound	*T. brucei*		*L. major*	
	MIC ratio	GI_50_ ratio	MIC ratio	GI_50_ ratio
**1**	10	12.7	10	11.0
**2**	> 1	> 7.2	> 1	> 6.7
**3**	1	3.2	1	4.0
**4**	1	> 31.4	1	> 13.1
**5**	1	> 8.7	1	1
**6**	1	> 16.6	1	1
Suramin	> 100	> 2381	nd	nd
Amphotericin B	nd	nd	> 1000	1408

*MIC ratio* MIC_HL-60_/MIC_parasite_, *GI*_*50*_* ratio* GI_50,HL-60_/GI_50,parasite_, MIC ratios and GI_50_ ratios were calculated from MIC and GI_50_ values shown in Table 1

*nd* not determined

Compared with suramin and amphotericin B, two drugs used in the treatment of sleeping sickness and cutaneous leishmaniasis, respectively, the six limonoids were 10–1000 times less active (Table 1). The higher antiparasitic activity of the drugs is also reflected in their greater selectivity indices of at least > 100 (Table 2).

**Fig. 2:**
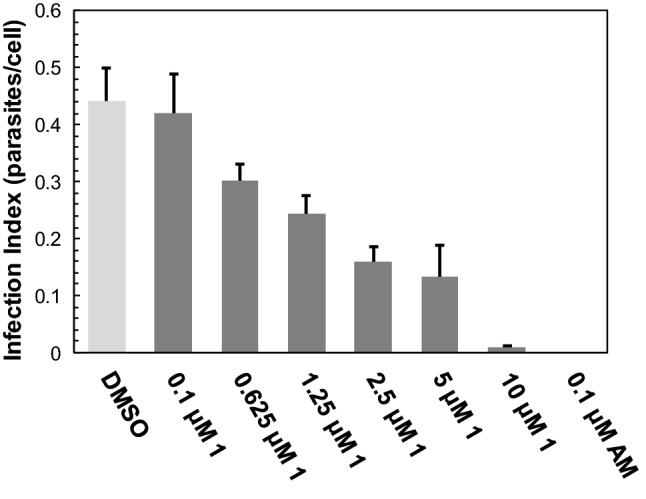
Effect of compound **1** on intracellular *L. major* amastigotes. After infection of J774 macrophages with metacyclic promastigotes of *L. major*, cells were treated with compound **1** or amphotericin B (AM) or with DMSO alone (DMSO) 24 h post infection. After 72 h incubation, coverslips with cells were washed with PBS, fixed with paraformaldehyde and stained with Giemsa. Subsequently, cells were destained and coverslips mounted, and the infection index determined. Mean values ± SD of three independent experiments run in triplicate are shown

As compound **1** was found to be the most potent leishmanicidal limonoid, we investigated its ability to kill *L. major* amastigotes within macrophages. The compound was able to reduce the parasite burden of infected macrophages with an ED_50_ (50% effective dose) of 1.51 μM (Fig. 2). Thus, compound **1** was almost twice as effective in affecting intracellular amastigotes than extracellular promastigotes (GI_50(promastigotes)_ = 2.86 μM vs ED_50(amastigotes)_ = 1.51 μM). At 10 μM, compound **1** killed completely intracellular amastigotes and only vacuoles containing fragments of parasites could be seen (Supplementary Figure S7). However, compared with amphotericin B, compound **1** was less potent in killing amastigotes within macrophages. The licensed drug was able to completely destroy intracellular amastigotes at a concentration of 0.1 μM (Fig. 2).

The trypanocidal and leishmanicidal activity of compounds **1**–**6** are in good agreement with previously reported antiprotozoal activities. For instance, the GI_50_ values for their anti-plasmodial activity were found to be within the range of 1.7–19.3 μM [8]. Compounds **3** and **4** have been previously reported to inhibit the growth of bloodstream forms of *T. brucei rhodesiense* with GI_50_ values of 3.4 μM [21]. Axenic grown amastigotes of *L. donovani* were affected by **3** and **4** with GI_50_ values of around 1 μM [21]. These activities are somewhat lower than those we found for **3** and **4** with promastigotes of *L. major*. The observed differences may be due to that axenic grown amastigotes are more sensitive as they are removed from their natural environment of a host cell.

Structure–activity relationship analysis revealed that the presence of a hydroxyl group at the C-7 position reduces the activity of the gedunin-type limonoids. For example, compound **2** with a hydroxyl group at C-7 displayed 5–6 times lower trypanocidal and leishmanicidal activity than compound **1** with a hydrogen atom at the position (Fig. 1 and Table 1). Likewise, compound **4** is 1.5–4.6 times less active than compound **3** which has a keto functional group instead of a hydroxyl group at the C-7 position (Fig. 1 and Table 1). These findings indicate that a slight increase in hydrophilicity reduces the trypanocidal and leishmanicidal activity of the gedunin-type limonoids.

This study has shown that phytochemical investigation of plants utilised in traditional medicine can yield the identification of compounds apparently responsible for the activity ascribed to their indigenous use. Here it was revealed that six limonoids isolated form the roots of *P. kotschyi*, a plant used for treating trypanosomiasis in domestic animals in Nigeria [6], display anti-trypanosomal activity. Kotschyienone A (**1**) was found to be not only the most active compound against *T. brucei* but also against *L. major*. Whereas compound **1** did not fully match the GI_50_ activity criteria for drug candidates for African trypanosomiasis [< 0.2 μg/mL vs 1.09 μg/mL (2.48 μM)], it fulfilled the GI_50_ activity criteria for drug candidates for leishmaniasis [< 1 μg/mL vs 0.66 μg/mL (1.51 μM)] [22]. The moderate selectivity indices of compound **1** did not correspond with selectivity index activity criteria for both parasite species (*Trypanosoma* > 100; *Leishmania* > 20 [22]). However, it should be pointed out that in this study a cancer cell line was used for determining selectivity and that, compared with non-malignant cells, the cytotoxicity of compound **1** may, therefore, be overestimated. For example, the cytotoxicity of **1** for the immortalised human embryonic kidney cell line HEK239T was previously determined to be > 200 μg/mL [8]. Based on this cytotoxicity, compound **1** would match the selectivity index activity criteria for drug candidates for both bloodstream forms of *T. brucei* and intracellular amastigotes of *L. major*. In conclusion, the gedunin-type limonoids seem to be a promising class of compounds for further anti-trypanosomal and anti-leishmanial drug development.

## Electronic supplementary material

Below is the link to the electronic supplementary material.

Supplementary file 1 (DOCX 3337 kb)
