# Supplementary material for: Trypanocidal and leishmanicidal activity of six limonoids
Source: J Nat Med. 2020 Apr 10;74(3):606–11. doi: 10.1007/s11418-020-01408-7 (PMC7253382; doi:10.1007/s11418-020-01408-7)
Supplement: Supplementary file 1 — (DOCX 3337 kb) [file 11418_2020_1408_MOESM1_ESM.docx]

SUPPLEMENTARY MATERIAL

**Trypanocidal and leishmanicidal activity of six limonoids**

**Dietmar Steverding^1^ • Lazare S. Sidjui^2,3^ • Éden Ramalho Ferreira^4,5^ • Bathelemy Ngameni^6^ • Gabriel N. Folefoc^3^ • Valérie Mahiou-Leddet^7^ • Evelyne Ollivier^7^ • G. Richard Stephenson^8^ • Thomas E. Storr^8^ • Kevin M. Tyler^4^**

- Dietmar Steverding

dsteverding@hotmail.com

- Bathelemy Ngameni

bath_ngameni@yahoo.fr

^1^ Bob Champion Research & Education Building, Norwich Medical School, University of East Anglia, Norwich NR4 7UQ, U.K.

^2^ Institute of Medical Research and Medicinal Plant Studies, P.O. Box 13033, Yaoundé, Cameroon

^3^ Bioorganic and Medicinal Chemistry Laboratory, Department of Organic Chemistry, Faculty of Sciences, University of Yaoundé I, Yaoundé, Cameroon

^4^ BioMedical Research Centre, Norwich Medical School, University of East Anglia, Norwich NR4 7TJ, U.K.

^5^ Departamento de Microbiologia, Imunologia e Parasitologia, Escola Paulista de Medicina, Universidade Federal de São Paulo, São Paulo, Brazi

^6^ Department of Pharmacognosy and Pharmaceutical Chemistry, Faculty of Medicine and Biomedical Science, University of Yaoundé I, Yaoundé, Cameroon

^7^ Aix-Marseille University, Avignon University, CNRS, IRD, IMBE, FAC PHARM, Marseille, France

^8^ School of Chemistry, University of East Anglia, Norwich NR4 7TJ, U.K.


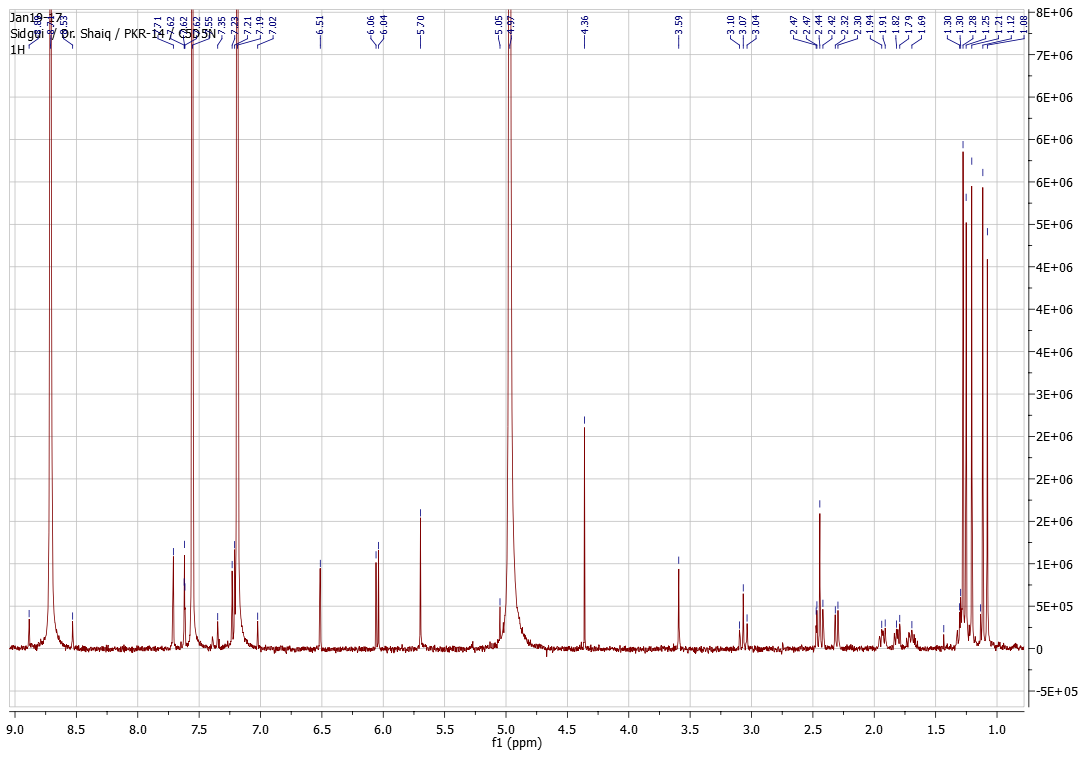

**Supplementary Figure S1.** ^1^H-NMR spectrum (top) and ^13^C-NMR spectrum (bottom) of compound **1**.


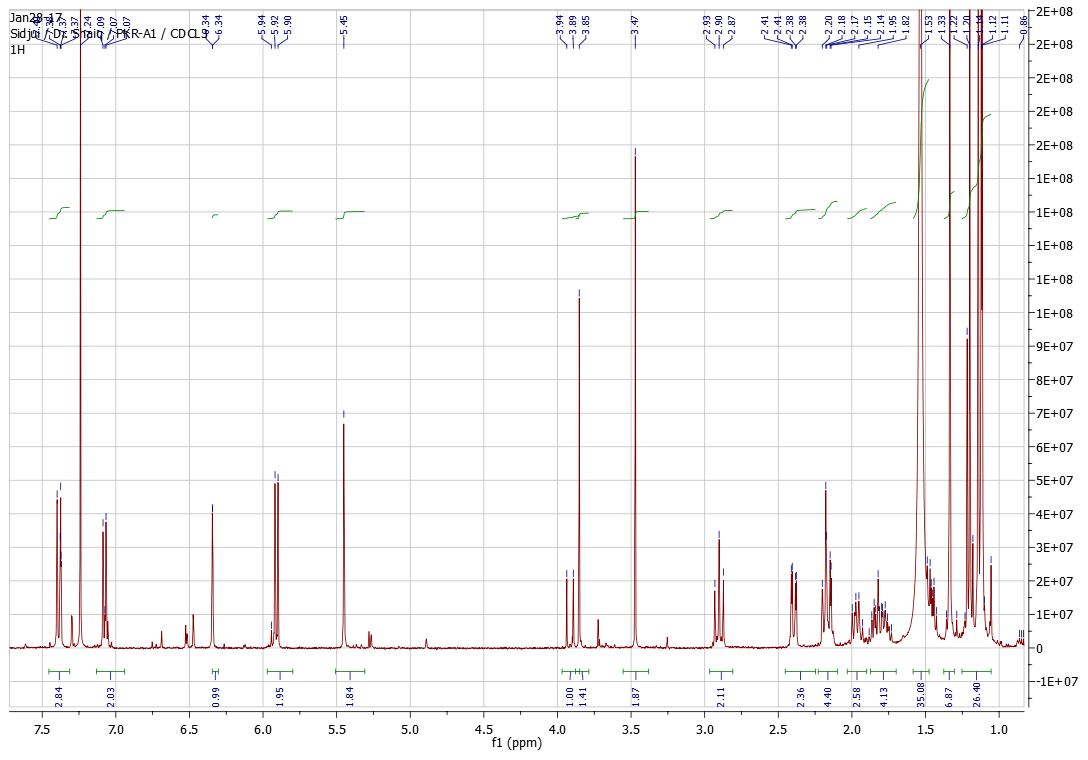


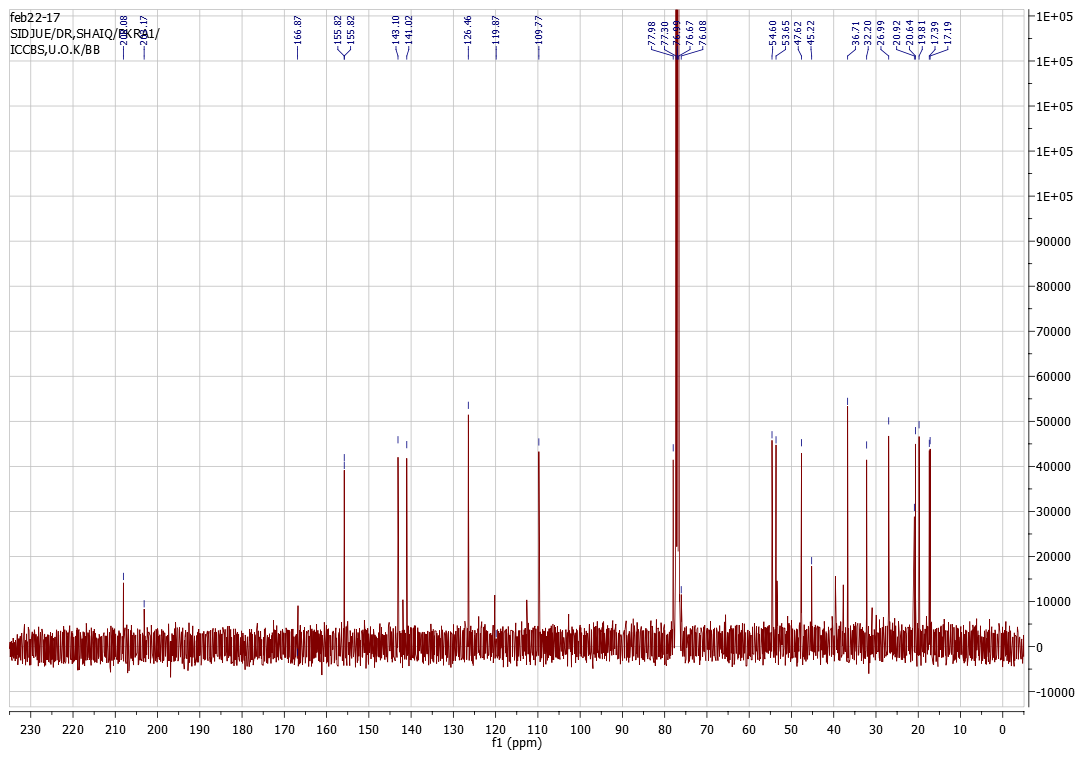


**Supplementary Figure S2.** ^1^H-NMR spectrum (top) and ^13^C-NMR spectrum (bottom) of compound **2**.

**
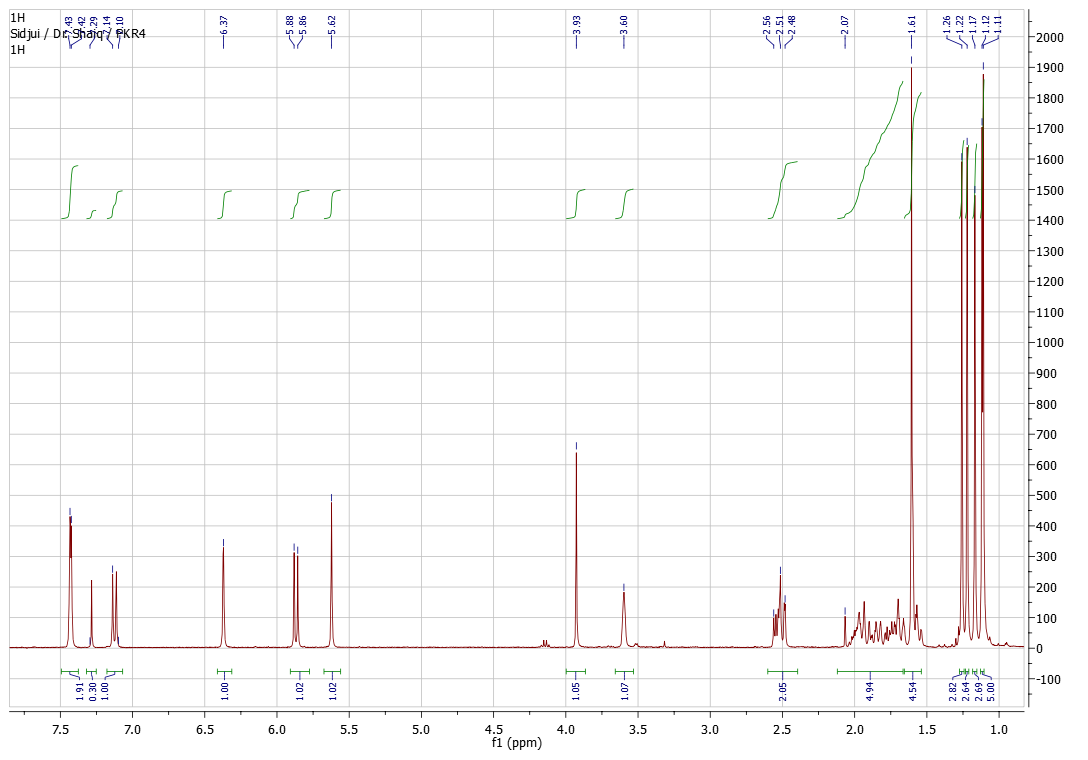
**

**
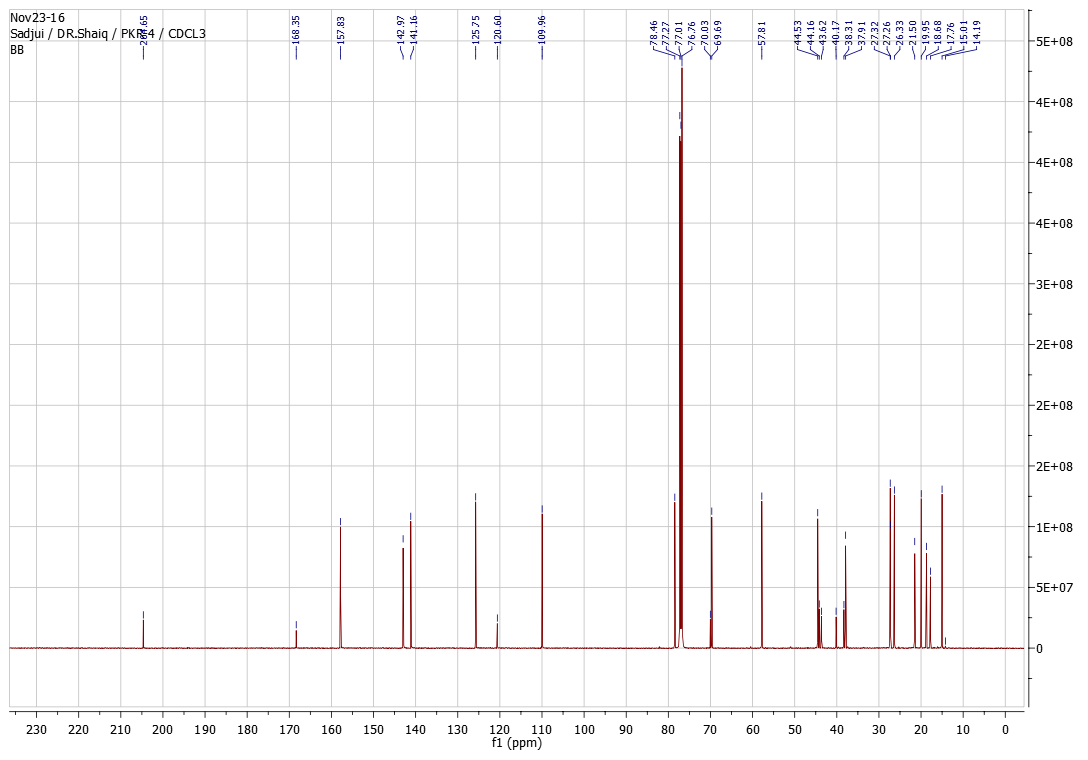
**

**Supplementary Figure S3.** ^1^H-NMR spectrum (top) and ^13^C-NMR spectrum (bottom) of compound **3**.

**
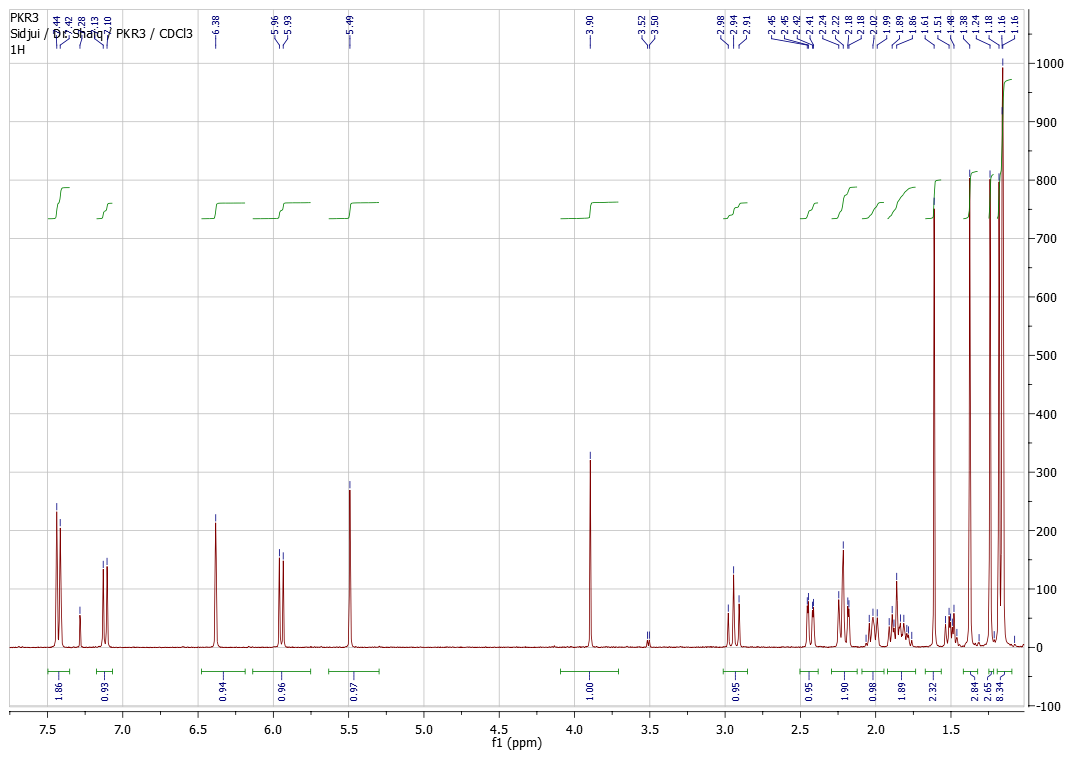
**

**
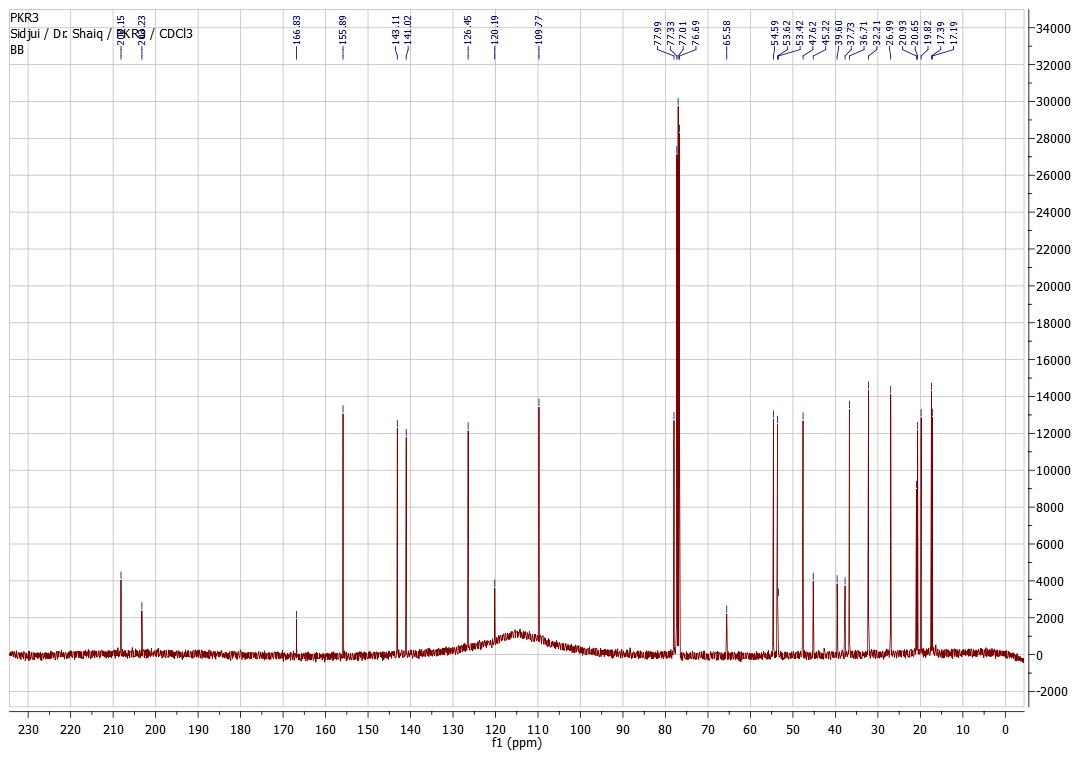
**

**Supplementary Figure S4.** ^1^H-NMR spectrum (top) and ^13^C-NMR spectrum (bottom) of compound **4**.

**
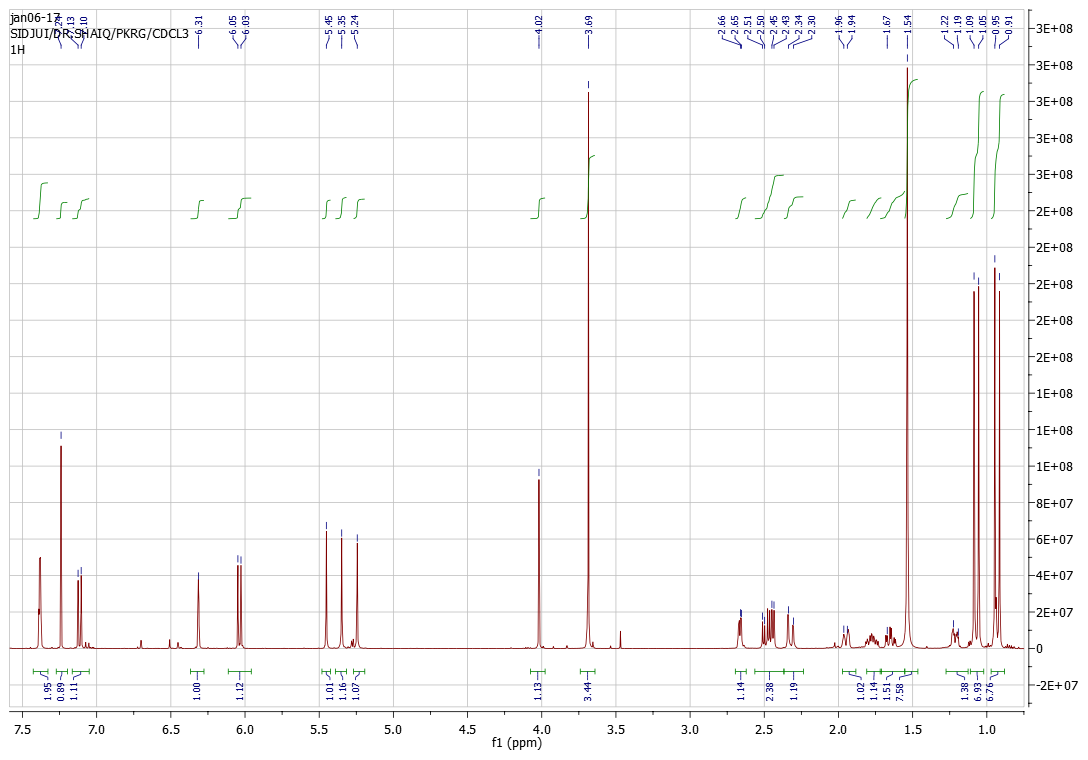
**


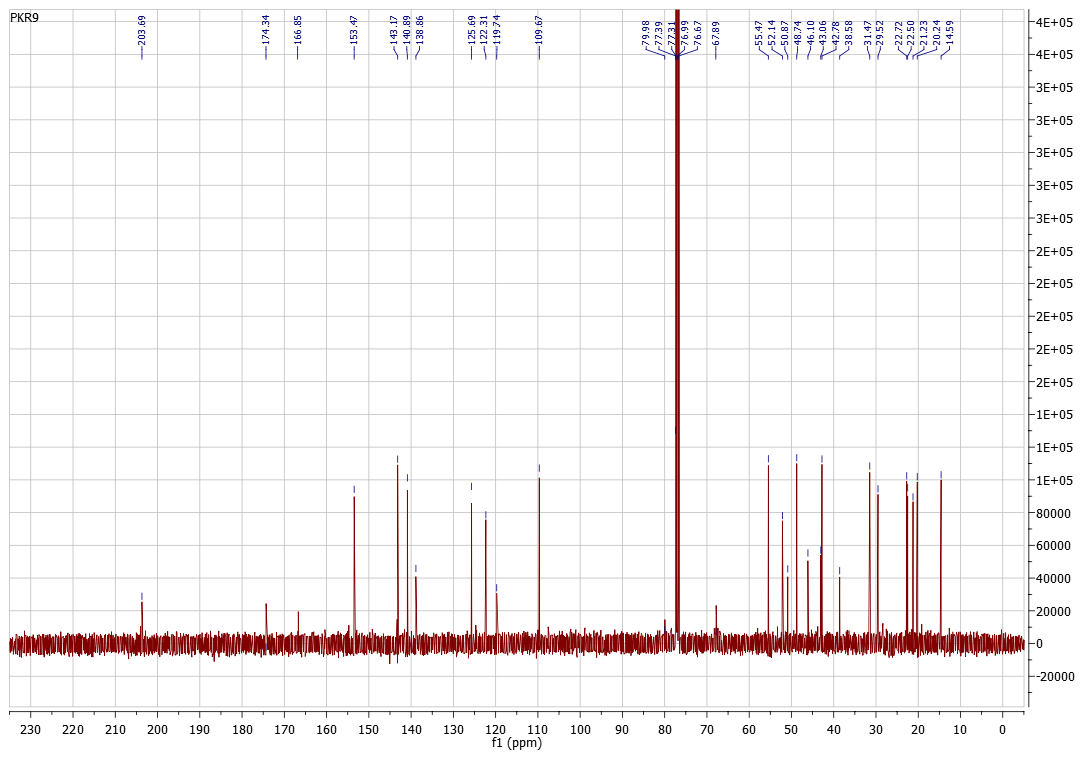


**Supplementary Figure S5.** ^1^H-NMR spectrum (top) and ^13^C-NMR spectrum (bottom) of compound **5**.

**
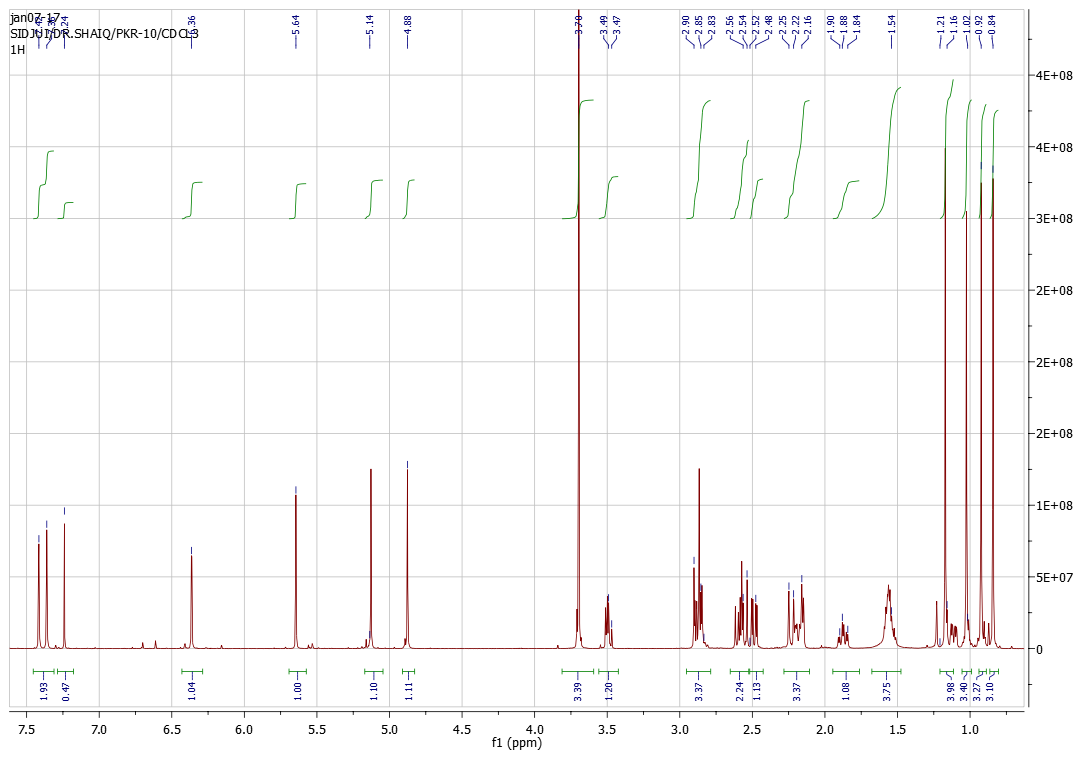
**

**
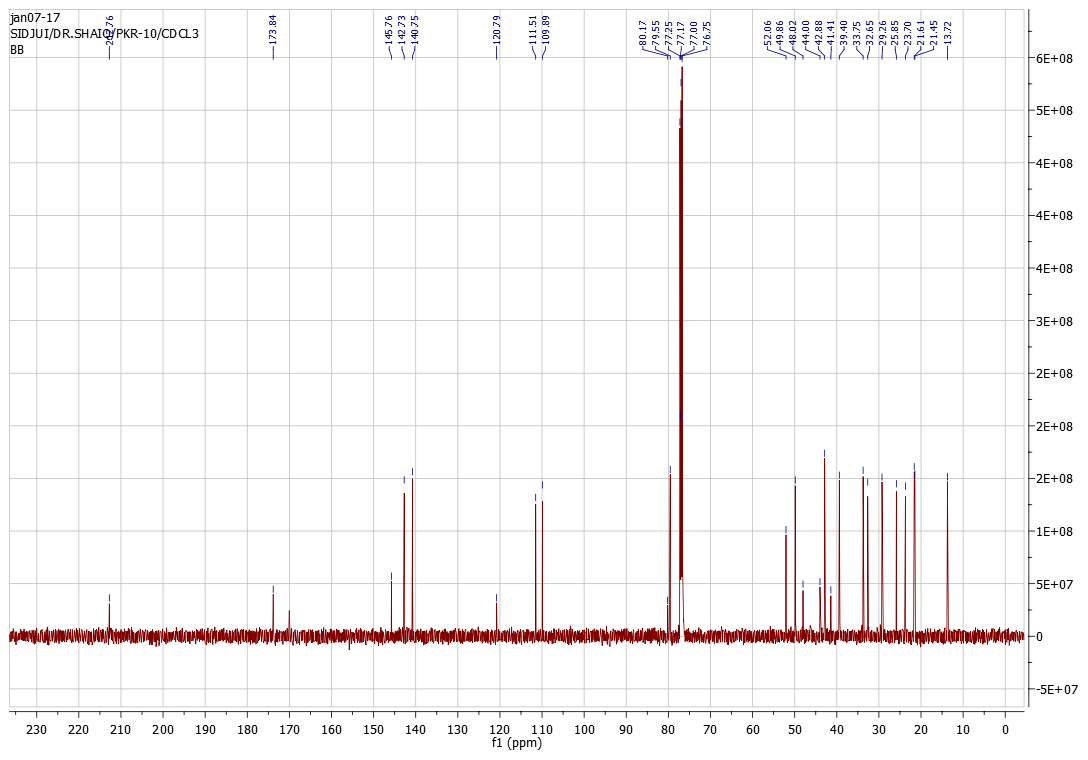
**

**Supplementary Figure S6.** ^1^H-NMR spectrum (top) and ^13^C-NMR spectrum (bottom) of compound **6**.


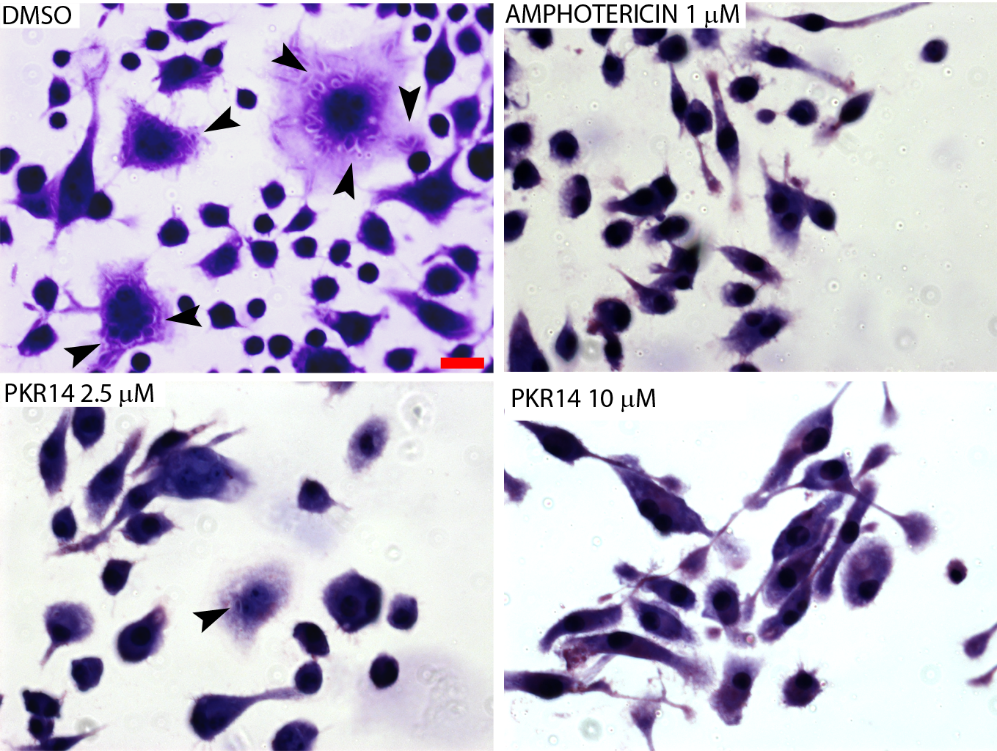


**Supplementary Figure S7.** Effect of compound **1** on intracellular *L. major* amastigotes. After infection of J774 macrophages with metacyclic promastigotes of *L. major*, cells were treated with DMSO, 1 μM amphotericin B, 2.5 μM compound **1** (PKR14 2.5 μM) or 10 μM compound **1** (PKR14 10 μM) 24 h post infection. After 72 h incubation, coverslips with cells were washed with PBS, fixed with paraformaldehyde and stained with Giemsa. Subsequently, cells were destained, coverslips mounted and photos taken using an Olympus BX50 microscope with a 100× N.A. 1.35 oil immersion objective equipped with a digital camera. Arrowheads indicate parasites within the macrophages. Red bar, 20 μm.
